# TTX-Bearing Planocerid Flatworm (Platyhelminthes: Acotylea) in the Ryukyu Islands, Japan

**DOI:** 10.3390/md16010037

**Published:** 2018-01-19

**Authors:** Hiroyuki Ueda, Shiro Itoi, Haruo Sugita

**Affiliations:** Department of Marine Science and Resources, Nihon University, Fujisawa, Kanagawa 252-0880, Japan; ueda.h.65039@gmail.com (H.U.); sugita.haruo@nihon-u.ac.jp (H.S.)

**Keywords:** flatworm, *Planocera*, planocerid, Polycladida, tetrodotoxin (TTX)

## Abstract

Polyclad flatworms comprise a highly diverse and cosmopolitan group of marine turbellarians. Although some species of the genera *Planocera* and *Stylochoplana* are known to be tetrodotoxin (TTX)-bearing, there are few new reports. In this study, planocerid-like flatworm specimens were found in the sea bottom off the waters around the Ryukyu Islands, Japan. The bodies were translucent with brown reticulate mottle, contained two conical tentacles with eye spots clustered at the base, and had a slightly frilled-body margin. Each specimen was subjected to TTX extraction followed by liquid chromatography with tandem mass spectrometry analysis. Mass chromatograms were found to be identical to those of the TTX standards. The TTX amounts in the two flatworm specimens were calculated to be 468 and 3634 μg. Their external morphology was found to be identical to that of *Planocera heda*. Phylogenetic analysis based on the sequences of the 28S rRNA gene and cytochrome-*c* oxidase subunit I gene also showed that both specimens clustered with the flatworms of the genus *Planocera* (*Planocera multitentaculata* and *Planocera reticulata*). This fact suggests that there might be other *Planocera* species that also possess highly concentrated TTX, contributing to the toxification of TTX-bearing organisms, including fish.

## 1. Introduction

Tetrodotoxin (TTX, C_11_H_17_N_3_O_8_), also known as pufferfish toxin, is one of the most potent neurotoxins. It specifically blocks voltage-gated sodium channels on excitable cell membranes of muscle and nerve tissues [1,2]. TTX was long believed to occur exclusively in pufferfish. However, after its molecular structure was found to be consistent with that of tarichatoxin from the California newt *Taricha torosa* [3], TTX has been detected in various taxonomic organisms including toad *Atelopus* spp. [4]; toxic goby *Yongeichthys criniger* [5]; blue-ringed octopus *Hapalochlaena maculosa* [6]; xanthid crabs [7]; marine bivalves [8]; gastropods [9]; flatworms [10]; and ribbonworms [11]. Furthermore, TTX-producing bacteria have been isolated from TTX-bearing organisms, as well as the environment [12,13]. In addition, non-toxic pufferfish were produced when grown from hatching with a non-toxic diet, and these cultured non-toxic pufferfish became toxic when TTX was administered orally [14,15,16,17,18,19,20]. Therefore, it is generally thought that TTX is produced primarily by bacteria, and accumulates in the pufferfish body via the food web [21,22]. Nevertheless, the source of TTX in pufferfish is actually unknown since the amount of TTX produced by the bacteria is too little to account for that present in pufferfish.

Recently, our lab showed that the pufferfish *Takifugu niphobles* ingested the toxic eggs of another pufferfish *Takifugu pardalis*, thereby efficiently increasing their own toxicity [23], suggesting that there may be other such unknown avenues of TTX accumulation. TTX accumulation in pufferfish via the ingestion of TTX-bearing organisms is also likely. Among the many TTX-bearing organisms listed above, *Planocera multitentaculata*, widely distributed in the waters around the Japanese Archipelago, was the first to be reported as a TTX-bearing flatworm [10]. Subsequently, TTX was detected in a closely related species, *Planocera reticulata* [24], and a conspecific [25]. Our lab recently found that the pufferfish of the genus *Takifugu* (*Takifugu rubripes* and *T. niphobles*) became toxic after feeding on the polyclad flatworm, *P. multitentaculata*, and a DNA fragment of the *P. multitentaculata* cytochrome-*c* oxidase subunit I (COI) gene was detected in the intestinal contents of wild specimens of the pufferfish *T. niphobles* (unpubl data). These results suggest that planocerid flatworms could contribute to the toxification of pufferfish. A different polyclad flatworm, *Stylochoplana* sp., has been implicated in the toxification of sea slugs, resulting in dog neurotoxicosis in New Zealand [26,27,28]. After a hiatus since the 1940s [29], these findings have spurred renewed interest in understanding these flatworms, with a spate of reports in the past decade [30,31,32,33,34].

Recently, our lab showed [35] that the classification of polyclads based on the 28S rRNA gene was approximately consistent with the morphological classification reported thus far [29,36,37]. In another recent study, we reported that the TTX content of the flatworm *P. multitentaculata* rose in association with an increase in body weight [38]. Almost nothing is known about how this increase takes place as there is little information on the diet or even the spatial (vertical or horizontal) distribution of the genus *Planocera* in the waters around the Japanese Archipelago. Only five species of this genus have been recorded from the area [29,36]. Of these, only two species, *P. multitentaculata* and *P. reticulata*, have been frequently observed at the intertidal zone of the main islands [29,38].

In this paper, to clarify the processes for the toxification of TTX-bearing organisms, including fish, we report the presence of planocerid-like flatworm specimens in the waters around the Ryukyu Islands, Japan, classify them with the help of phylogenetic analysis using 28S rRNA and COI nucleotide sequences, and measure the toxicity of the flatworm by means of liquid chromatography with tandem mass spectrometry (LC-MS/MS) analysis. Finally, we discuss the distribution of planocerid flatworms in Japan, and their contribution to the toxification of pufferfish.

## 2. Results

### 2.1. External Morphology

**Figure 1:**
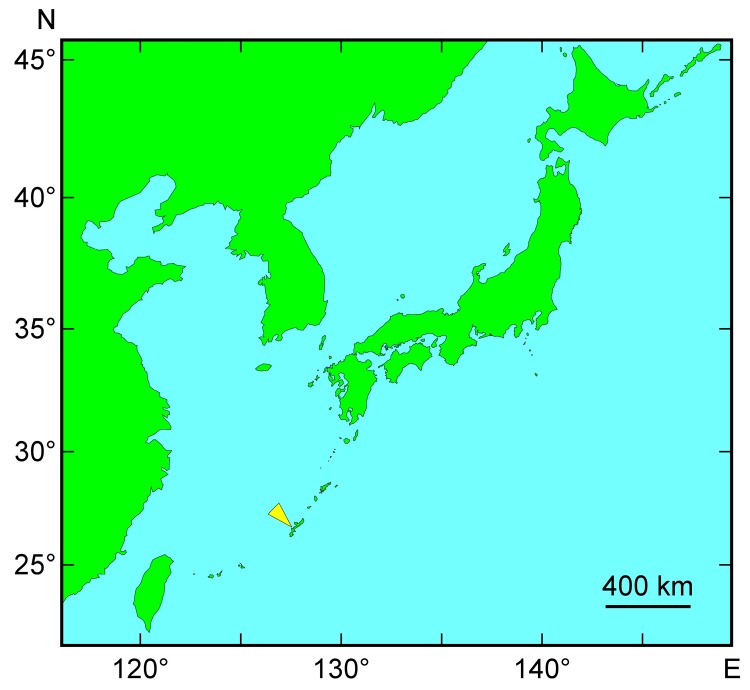
Sampling location for the polyclad flatworms used in this study (shown by a yellow arrow).

**Figure 2:**
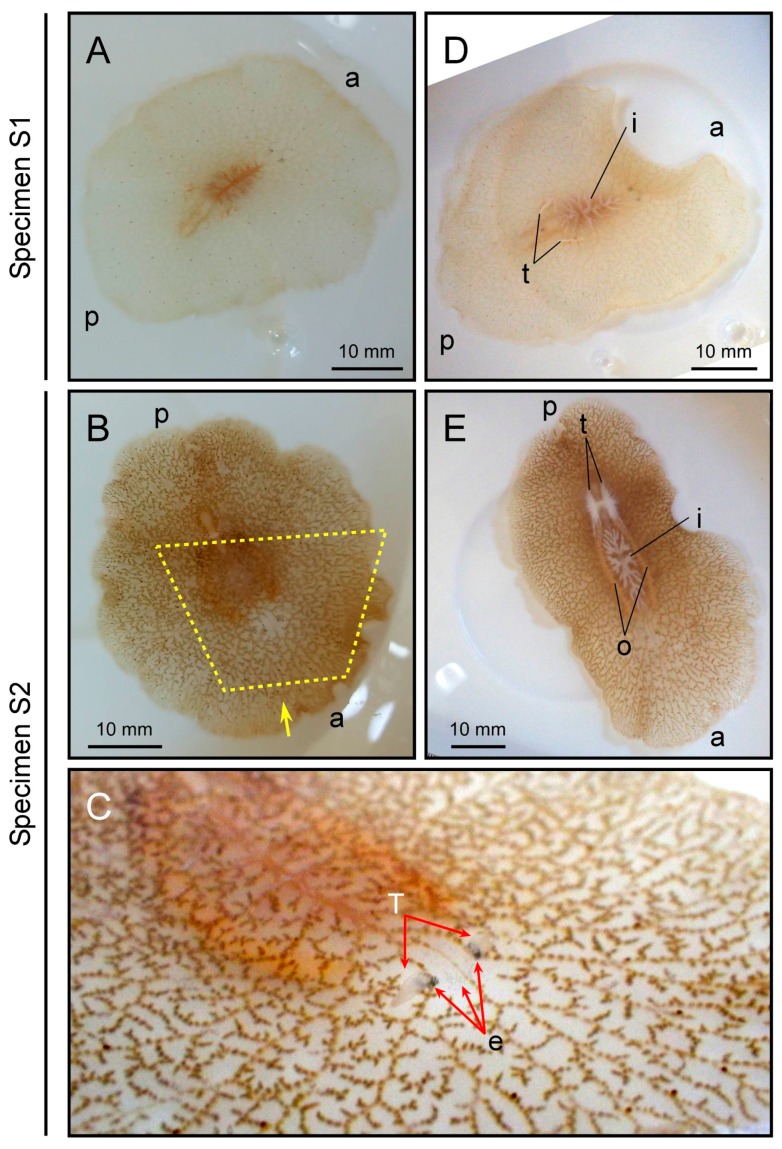
External morphology of polyclad flatworms. Upper and lower panels represent specimens S1 and S2, respectively. Panel (**A**,**B**) dorsal views; Panel (**C**) anterior region indicated by the yellow dotted line from the direction of the yellow arrow in panel (**B**); Panel (**D**,**E**) ventral views. a and p represent anterior and posterior ends of the body, respectively. t, testis; o, ovary; T, tentacle; e, eye spots; i, intestine.

Two polyclad flatworms were collected from waters off the city of Nago, Okinawa main island, Japan (Figure 1, Table 1), in August 2017. Specimen-1 (hereafter referred to as S1) weighed 1.88 g and Specimen-2 (S2) weighed 2.69 g. Both appeared to be planocerids based on external morphology, although some features were different from those of *P. multitentaculata* and *P. reticulata*, based on the only report so far [29]. Their external morphology was characterized by a translucent body with brown reticulate mottle (light in color for S1 and dark for S2; Figure 2A,B) with a large number of grey/faint red spots, two conical tentacles with eye spots clustered at the base (Figure 2C), and a slightly frilled-body margin (Figure 2A,B). Testes were observed in both specimens, while ovaries were only found in S2 and were deeply reticulate and maculate (Figure 2D,E).

### 2.2. Molecular Phylogenetic Inference and Taxonomy

**Figure 3:**
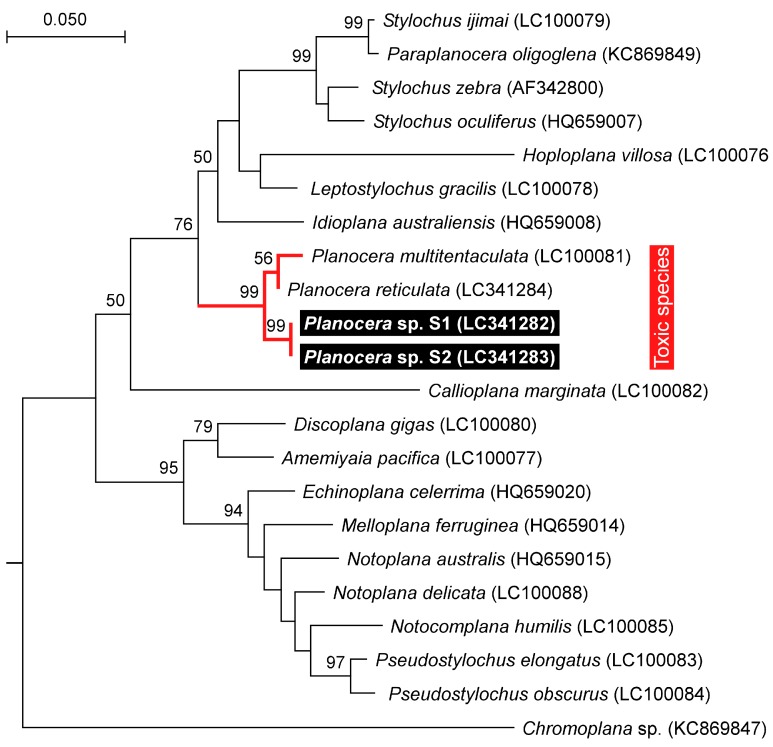
Phylogenetic relationship of the planocerid-like flatworms (*Planocera* sp.) and related polyclad species in Acotylea inferred from the 28S rRNA gene sequence. The phylogenetic tree was generated by maximum likelihood analysis. Numbers at branches denote the bootstrap percentages from 1000 replicates. The accession numbers for the sequences are shown in parentheses. The accession numbers LC341282–LC341284 refer to those deposited in the DDBJ/EMBL/GenBank databases in this study. The sequence from *Chromoplana* sp. was used as the outgroup. Only bootstrap values exceeding 50% are presented. The scale refers to nucleotide substitutions per site.

**Figure 4:**
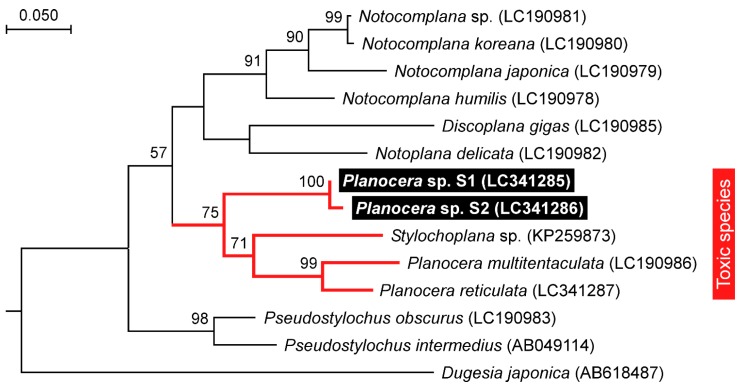
Phylogenetic relationship of the planocerid-like flatworms (*Planocera* sp.) and related polyclad species in Acotylea inferred from the COI gene sequence. The phylogenetic tree was generated by maximum likelihood analysis. Numbers at branches denote the bootstrap percentages from 1000 replicates. The accession numbers for the sequences are shown in parentheses. The accession numbers LC341285–LC341287 refer to those deposited in the DDBJ/EMBL/GenBank databases in this study. The sequence from *Dugesia japonica* was used as the outgroup. Only bootstrap values exceeding 50% are presented. The scale refers to nucleotide substitutions per site.

The 28S rRNA (approximately 1100 bp) and COI (approximately 750 bp) gene fragments from both specimens were sequenced and subjected to phylogenetic analysis along with orthologous sequences from all the other Acotylean flatworms reported so far [35]. The resulting maximum likelihood trees from both sequences showed that S1 and S2 clustered with the toxic flatworms, *P. multitentaculata* and *P. reticulata*, separately from the non-toxic flatworms in the 28S rRNA tree, with high bootstrap support (Figure 3 and Figure 4). A COI tree including the sequence of *Stylochoplana* sp. [27] also showed an apparent toxicity-based clustering of flatworms, with *P. multitentaculata* and *P. reticulata* co-occurring with *Stylochoplana* sp. (Figure 4). The DNA sequences of the 28S rRNA and COI gene fragments from this study have been submitted to the DDBJ/EMBL/GenBank databases under the accession numbers LC341282–LC341284 and LC341285–LC341287, respectively.

### 2.3. Toxicity

**Figure 5:**
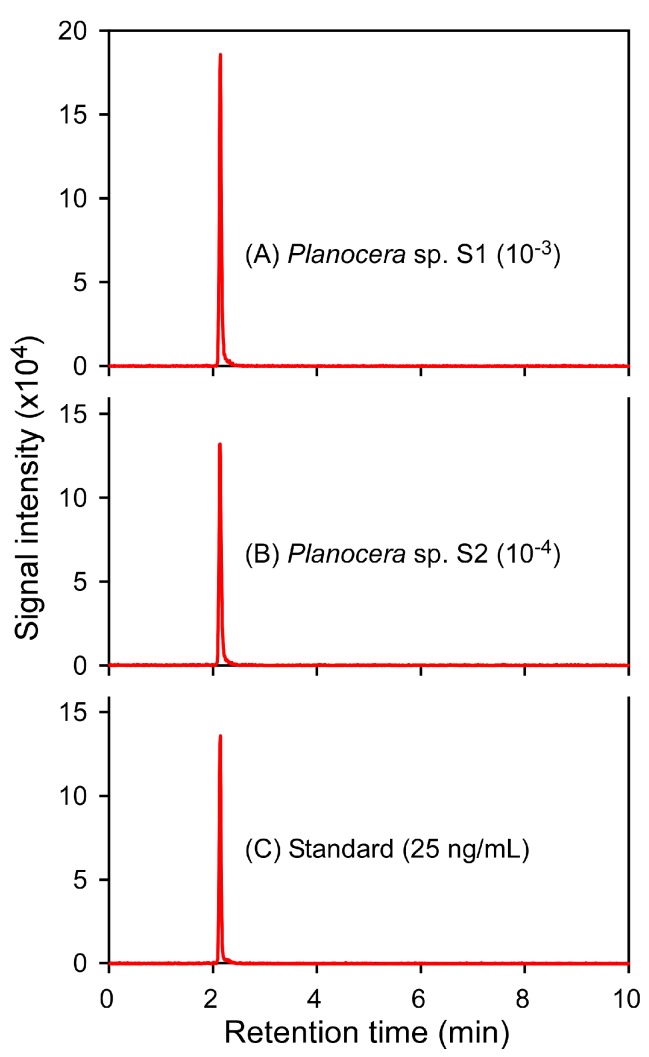
Mass chromatograms of the LC-MS/MS obtained under MRM mode (*m*/*z* 320 > 302). MRM patterns of the extract from specimen S1 (**A**), the extract from specimen S2 (**B**), and 25 ng/mL TTX standard (**C**). The MRM patterns were obtained from S1 and S2, with 1000- and 10,000-fold dilution, respectively.

The two flatworms were subjected to TTX extraction followed by LC-MS/MS analysis. Mass chromatogram of the LC-MS/MS was obtained under the multiple-reaction monitoring (MRM) mode, with detection in positive mode, and analysis of two product ions at *m*/*z* 162 for the quantification of TTX and *m*/*z* 302 for confirmation of the compound from the precursor ion at *m*/*z* 320 (Figure S1), and the MRM patterns of the flatworms were found to be identical to those of the TTX standards (Figure 5). No effect on the actual yield was observed during the process of TTX extraction using 0.1% acetic acid (*v*/*v*) with mechanical filtration: no significant difference in the signal intensities was observed for samples before and after filtration, and almost all the TTX added to the flatworm tissues was recovered in the extraction process. A calibration curve generated using 1–100 ng/mL TTX standards showed good linearity and precision (*y* = 119.609*x* − 34.773, *R*^2^ = 0.9897), where LOD was 15.45 ng/mL. The TTX concentration in S1 and S2 was calculated measuring extracts with 1000- and 10,000-fold dilution, respectively, to be 249 and 1351 μg/g (which corresponding to 1132 and 6141 MU/g, respectively), and therefore, the total amount of TTX in the body was calculated to be 468 and 3634 μg, corresponding to 2127 and 16,518 MU, respectively (Table 1).

## 3. Discussion

TTX has previously been detected in several flatworms such as the genera *Planocera* [10,24,25] and *Stylochoplana* [27,28]. *Stylochoplana* was shown to be involved in dog neurotoxicosis through toxification of the sea slug *Pleurobranchaea maculata* with TTX on some New Zealand beaches [26,27,28]. Thus far, however, the mechanism of toxification of pufferfish has remained unclear. Although it is generally accepted that pufferfish accumulates TTX in the body through the food web, other means have also been suggested since in vivo cultured TTX-producing bacteria are unable to produce enough quantities of TTX to account for the amount of TTX in wild pufferfish [39,40,41,42]. In the past, flatworms were raised as candidates for the source of toxification of the pufferfish *T. rubripes*, but the level of toxification was low [43], and there has not been much investigation since. Among the TTX-bearing flatworm species, *P. multitentaculata* is rather large, and the amount of TTX rises in association with body size [38]. Additionally, *P. reticulata*, a closely related species of *P. multitentaculata*, also possesses a large concentration of TTX [24], and their geographical distributions overlap [29]. In addition, the geographical distributions of both flatworm species are consistent with those of TTX-bearing fishes including toxic pufferfish from the genus *Takifugu* [44]. However, no report on the distribution of planocerid flatworms is currently available for the waters around the Ryukyu Islands, which harbor many TTX-bearing organisms, including fish, such as toxic pufferfish *Chelonodon* spp. [45], and toxic goby *Y. criniger* [46].

The flatworm specimens in this study appear to clearly belong to the genus *Planocera* based on external morphology and phylogenetic analysis. Five records are available as accepted species in the genus *Planocera* including *P. multitentaculata* and *P. reticulata* from the waters around the Japanese Archipelago [29,36]. Several external characteristics of the flatworms in this study were consistent with those of *Planocera heda*, which was captured from the west coastal waters off the Izu Peninsula (34°58′28″ N, 138°46′21″ E), Japan, using the dredge. *P. heda* was classified and described by Kato [29], based on only one specimen. As in the case of *P. heda*, the flatworms in this study were also collected from the sea bottom, at a depth of approximately 10 m off Nago, Okinawa, Japan, which is more than 1400 km from the Izu Peninsula, Japan. Unfortunately, there is no way at present to confirm whether our specimens are *P. heda*, because the type specimen was lost during World War II. Nevertheless, it appears very likely that the flatworm specimens in this study are either *P. heda* or closely related species.

Phylogenetic analysis based on the sequence of the COI gene fragment showed that *Stylochoplana* sp., the flatworm species that contains a large amount of TTX, clustered with those from the genus *Planocera* and its close relatives. This result suggests that the common ancestor of the genus *Planocera* and its related species acquired the ability to accumulate TTX in its body. However, further investigations on this subject are clearly warranted.

In the present study, we showed that the concentration and amount of TTX of both our specimens were comparable to those in the toxic flatworm *P. multitentaculata*. As with the seasonal changes in the amount of TTX in *P. multitentaculata* [38], it is expected that the flatworms in this study also possess a larger amount of TTX in the spawning season in association with the increased body size: indeed, the concentration and amount of TTX were higher in the specimen with ovaries and testes than in that with testes only, suggesting that the eggs and larvae contain highly concentrated TTX, as in the case of *P. multitentaculata* [38]. Additionally, TTX content of the planocerid flatworms in this study might rise in association with an increase in body weight, as in the case of *P. multitentaculata* [38]. These flatworms are expected to be in an important supplier of TTX to TTX-bearing organisms, including fish, in the waters around the Ryukyu Islands. It will be worthwhile to invest in the detailed study of the available amount of TTX resource and ecology of the flatworms.

## 4. Materials and Methods 

### 4.1. Flatworm Individuals

Planocerid-like flatworms were collected from underneath rocks on the inshore sandy bottom at a depth of approximately 10 m off Nago, Okinawa, Japan (Figure 1), by SCUBA diving. Each specimen was photographed and its external features recorded, after which a small portion of the body tissue was excised for DNA sequencing. The rest of the specimen was stored at −20 °C until TTX extraction, detection, and quantification.

### 4.2. DNA Extraction and Polymerase Chain Reaction Amplification

Total genomic DNA was extracted from the excised tissue sample for each specimen using the method of Tsunashima et al. [35] with some modifications. Briefly, proteinase K-treated samples were subjected to phenol/chloroform extraction with MaXtract High Density (Qiagen, Germantown, MD, USA) following the manufacturer’s protocol. Partial fragments of the 28S rRNA gene (approximately 1100 bp), including the D1–D2 region, were amplified by PCR using the universal primers HRNT-F2 (5′-AGTTC AAGAG TACGT GAAAC C-3′) and HRNT-R2 (5′-AACAC CTTTT GTGGT ATCTG ATGA-3′) [35]. Partial fragments of the mitochondrial gene (approximately 750 bp), COI, were amplified by PCR using the universal primers HRpra2 (5′-AATAA GTATC ATGTA RACTD ATRTC T-3′) and HRprb2-2 (5′-GDGGV TTTGG DAATT GAYTA ATACC TT-3′) [35]. The reaction mixture for PCR amplification contained genomic DNA as a template, 0.625 units of *TaKaRa ExTaq* DNA polymerase (Takara Bio, Otsu, Shiga, Japan), 2 μL of 10× *ExTaq* DNA polymerase buffer (Takara Bio), 2.6 μL of 10 μM primers, 1.6 μL of 2.5 mM dNTP, and sterile water to bring the total volume up to 20 μL. PCR was done with an initial denaturation at 95 °C for 1 min followed by 35 cycles of denaturation at 95 °C for 10 s, annealing at 50 °C for 30 s, and extension at 72 °C for 2 min.

### 4.3. Sequencing and Phylogenetic Analysis

Both strands of the PCR products were directly sequenced with a 3130*xl* Genetic Analyzer (Applied Biosystems, Foster, CA, USA) using a BigDye Terminator v3.1 Cycle Sequencing Kit (Applied Biosystems). 28S rRNA gene sequences for the following species were obtained from the DDBJ/EMBL/GenBank databases: *Amemiyaia pacifica* (LC100077), *Callioplana marginata* (LC100082), *Discoplana gigas* (LC100080), *Echinoplana celerrima* (HQ659020), *Hoploplana villosa* (LC100076), *Idioplana australiensis* (HQ659008), *Leptostylochus gracillis* (LC100078), *Melloplana ferruginea* (HQ659014), *Notocomplana humilis* (LC100085), *Notoplana australis* (HQ659015), *Notoplana delicata* (LC100088), *P. multitentaculata* (LC100081), *Paraplanocera oligoglena* (KC869849), *Pseudostylochus elongatus* (LC100083), *Pseudostylochus obscurus* (LC100084), *Stylochus ijimai* (LC100079), *Stylochus oculiferus* (HQ659007), and *Stylochus zebra* (AF342800). COI nucleotide sequences for the following species were also obtained from the databases: *D. gigas* (LC190985), *N. humilis* (LC190978), *Notocomplana japonica* (LC190979), *Notocomplana koreana* (LC190980), *Notocomplana* sp. (LC190981), *N. delicata* (LC190982), *P. multitentaculata* (LC190986), *Pseudostylochus intermedius* (AB049114), *P. obscurus* (LC190983) and *Stylochoplana* sp. (KP259873). *Chromoplana* sp. (28S rRNA: KC869847) and *Dugesia japonica* (COI: AB618487) were used as the respective outgroup species for the 28S and COI trees.

The nucleotide sequences of the partial 28S rRNA and COI genes for all the flatworms were aligned using Clustal Omega [47] with those in the DDBJ/EMBL/GenBank databases obtained using a BLAST search [48]. The alignment was then subjected to phylogenetic inference by means of the maximum likelihood method using MEGA ver. 6.0.6 [49].

### 4.4. LC-MS/MS Analysis

As described in an earlier paper from our lab [50], TTX was extracted from samples using 0.1% acetic acid. The extract was then filtered through a membrane of pore size 0.45 μm (SupraPure Syringe Filter, PTEE-Hydrophilic, Recenttec, Taipei, Taiwan) and subjected to analysis by LC-MS/MS. Quantification of the TTX was performed using a Quattro Premier XE (Waters, Milford, MA, USA) equipped with an electrospray ionization (ESI) source coupled to an Acquity UPLC system (Waters) [23]. Chromatographic separation was achieved using an Atlantis HILIC Silica column (2.1 mm × 150 mm, 5 μm; Waters), coupled to an Atlantis HILIC Silica pre-column (2.1 mm × 10 mm, 5 μm; Waters), with gradient elution using formic acid/acetonitrile. The mass spectrometer was operated in MRM mode, with detection in positive mode, and analysis of two product ions at *m*/*z* 162 for quantification of TTX and *m*/*z* 302 for confirmation of the compound from the precursor ion at *m*/*z* 320. A calibration curve was generated using 1 to 100 ng/mL of a TTX standard (Wako Pure Chemicals, Osaka, Japan), which showed good linearity and precision. Quantification of TTX was carried out using the data for samples with >1000-fold dilution to remove any matrix effect, since it was recovered from the samples with >1000-fold dilution. One mouse unit (MU) was defined as the amount of toxin required to kill a 20 g male ddY strain mouse within 30 min after intraperitoneal administration, and equivalent to 0.22 µg of TTX, based on the specific toxicity of TTX [51].

## 5. Conclusions

In summary, planocerid-like flatworms were found in the sea bottom of the waters around the Ryukyu Island, Japan. Their external morphology is similar to that of *P. heda*. Phylogenetic analysis based on the nuclear and mitochondrial genome sequences also showed that the flatworms formed a cluster with species from the genus *Planocera*. In addition, they possessed a large amount of TTX in their body, suggesting that these planocerid-like flatworms could be suppliers of the toxin to the TTX-bearing organisms such as pufferfish and toxic goby in the waters around the Ryukyu Islands.

## Figures and Tables

**Table 1:** Toxicity of planocerid flatworms used in this study. COI, cytochrome-*c* oxidase subunit I.

Specimen	Body Weight (g)	TTX Concentration (μg/g)	TTX Amount (μg)	Sequence	
				28S rRNA	COI
*Planocera* sp. S1	1.88	249	469	LC341282	LC341285
*Planocera* sp. S2	2.69	1351	3635	LC341283	LC341286

## Supplementary Materials

The following are available online at www.mdpi.com/1660-3397/16/1/37/s1, Figure S1: LC-MS/MS patterns of the TTX from planocerid flatworms specimen S1 (A) and S2 (B), and 25 ng/mL TTX standard (C).
