# Supplementary material for: TTX-Bearing Planocerid Flatworm (Platyhelminthes: Acotylea) in the Ryukyu Islands, Japan
Source: Mar Drugs. 2018 Jan 19;16(1):37. doi: 10.3390/md16010037 (PMC5793085; doi:10.3390/md16010037)
Supplement: Supplementary file 1 [file marinedrugs-16-00037-s001.pdf]

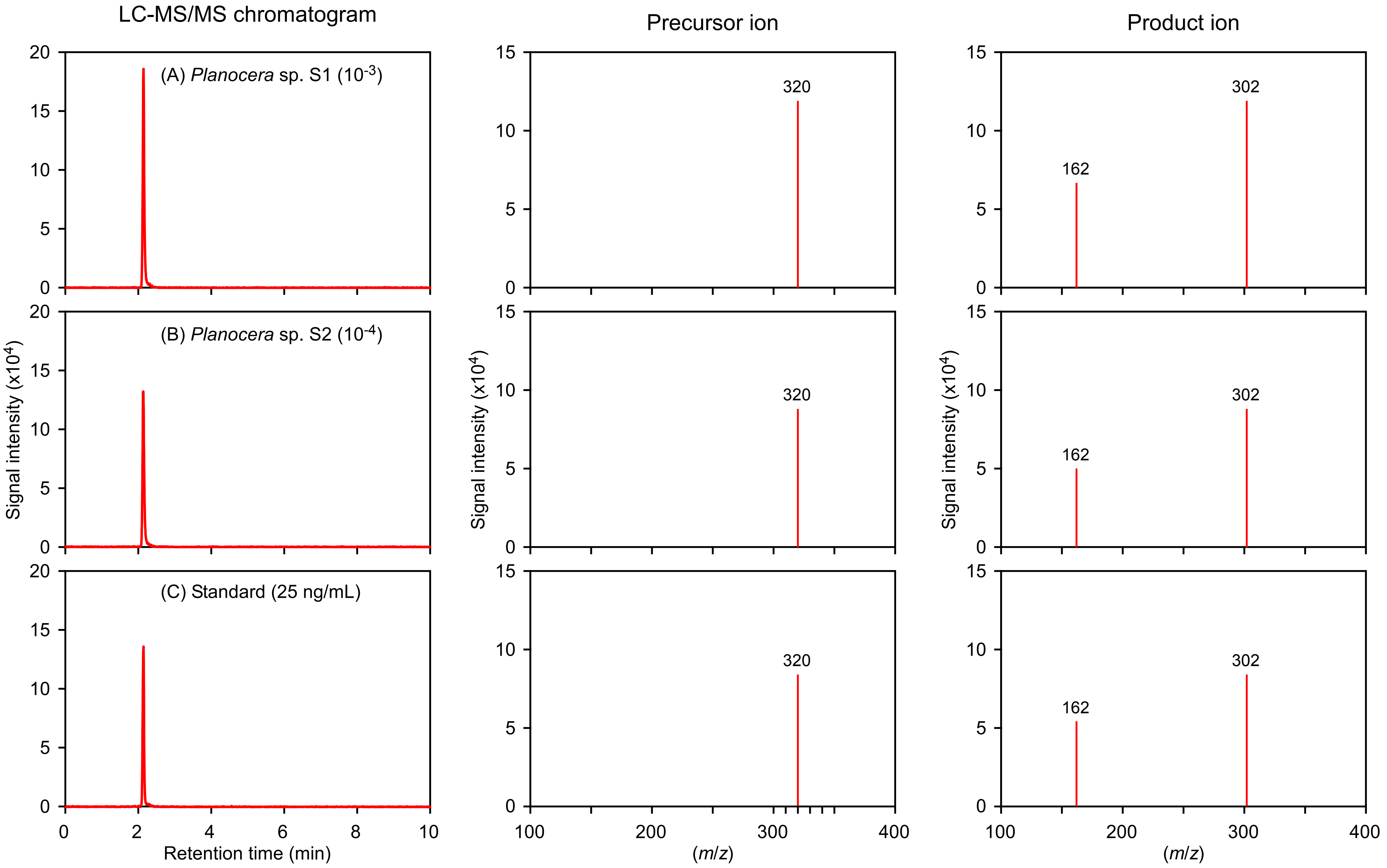

Figure S1. LC-MS/MS patterns of the TTX from planocerid flatworms specimen S1 (A) and S2 (B), and 25 ng/mL TTX standard (C). Left panels represent LC-MS/MS chromatograms, while the middle and right panels represent the mass spectrum of the precursor and product ions, respectively.
